# A mosquito-inspired theoretical framework for acoustic signal detection

**DOI:** 10.1073/pnas.2500938122

**Published:** 2025-09-05

**Authors:** Justin Faber, Alexandros C. Alampounti, Marcos Georgiades, Joerg T. Albert, Dolores Bozovic

**Affiliations:** ^a^Department of Physics and Astronomy, University of California, Los Angeles, CA 90095; ^b^University College London Ear Institute, London WC1X 8EE, United Kingdom; ^c^Department for Neuroscience, Cluster of Excellence Hearing4all, Sensory Physiology & Behaviour Group, School of Medicine and Health Sciences, Carl von Ossietzky Universität Oldenburg, Oldenburg 26129, Germany; ^d^California NanoSystems Institute, University of California, Los Angeles 90095, CA

**Keywords:** mosquito, auditory system, distortion product, signal detection, Hopf bifurcation

## Abstract

Distortion products are tones produced through nonlinear effects of a system simultaneously detecting two or more frequencies. These combination tones are ubiquitous to vertebrate auditory systems and are generally regarded as byproducts of nonlinear signal amplification. It has previously been shown that several species of infectious-disease-carrying mosquitoes utilize these distortion products for detecting and locating potential mates. Furthermore, the mechanical tuning curve of the male mosquito flagellum was shown not to be aligned with that of its sensory neural elements. Using a generic theoretical model for acoustic sensing, we evaluate the signal-detection advantages and disadvantages that are implied by these two schemes: distortion product detection and cascading a signal through multiple layers of oscillator elements of different characteristic frequency. Last, we show that the combination of these two schemes yields a signal detector with enhanced frequency selectivity and speed of response, thus enabling the detection of transient, narrowband flight tones.

## Significance

The simultaneous presentation of two acoustic tones can cause us to perceive additional tones at integer-multiple combinations of the two frequencies. These distortion products are ubiquitous to vertebrate ears and have even been measured in the auditory systems of mosquitoes. It has previously been shown that mosquitoes utilize distortion products for the acoustic detection of potential mates. In the current work, we propose a theoretical model of the mosquito auditory system to show that distortion products in conjunction with a cascade of signal amplifiers can actually give rise to significant improvement in signal detection. We speculate that this counterintuitive detection scheme may also be employed by other insects and could be of interest for the development of biologically inspired technology.

Mosquitoes pose a significant public health problem, as they spread pathogens that cause more than 700,000 deaths each year (1). Furthermore, predictions based on climate change models warn that the geographic areas which are vulnerable to mosquito-borne diseases such as malaria, dengue fever, and others, will greatly spread in the coming decades (2). Meanwhile, traditional means of controlling the mosquito population, utilizing chemicals such as DTT, have been decreasing in efficacy as resistance develops and have been proven to be detrimental to the environment (3). An understanding of the mechanisms of auditory detection in mosquitoes could enable alternative means of controlling mosquito populations, as the male mosquitoes rely on their sense of hearing to locate potential mates (4, 5). However, many basic questions remain open in our understanding of how a mosquito performs sensitive, rapid, and highly frequency-tuned detection of sound.

**Fig. 1.**
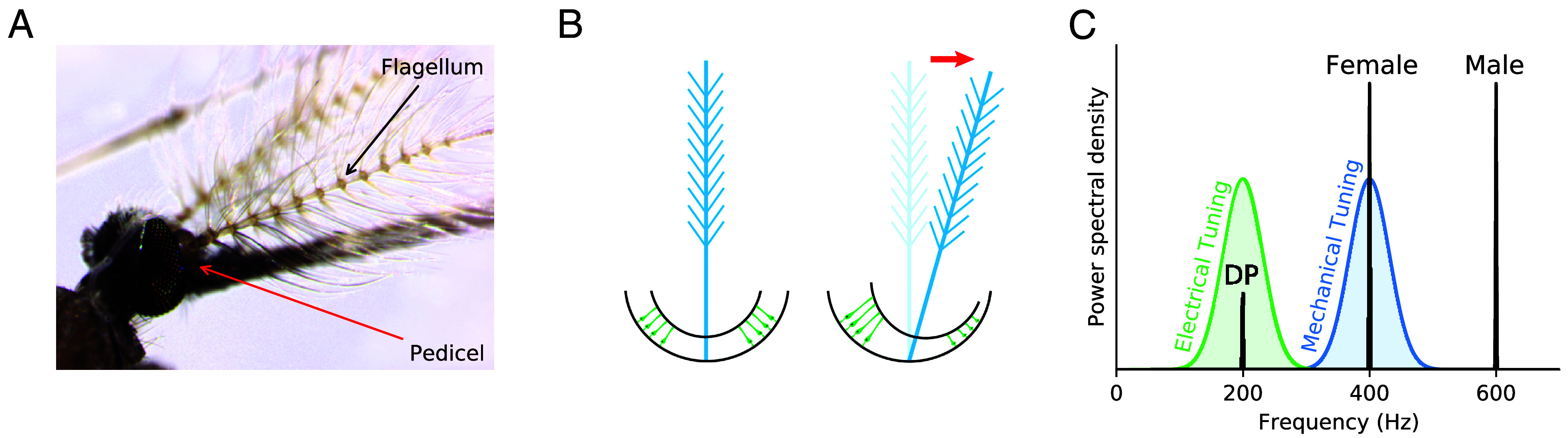
Mosquito hearing. (*A*) The feather-like flagellum and the bowl-shaped pedicel, which houses the Johnston’s organ (male *Anopheles gambiae*). (*B*) Illustration of the mechanical connection between the flagellum (blue) and the active sensory neurons (green). A deflection of the flagellum causes an extension of the sensory elements. (*C*) Approximate tuning curves of the mechanical components of the organ (blue) and the electrical response of the neurons (green) for male mosquitoes. Female and male wingbeat frequencies are indicated, along with a resulting distortion product (DP).

Despite their largely different structures, the auditory systems of insects exhibit several parallels to those of vertebrates, which have been much more extensively studied (6, 7). First, both systems have been shown to involve mechanoelectrical transduction, a process by which vibrations induced by the incoming sound wave cause mechanical gating of the associated ion channels, thus converting a mechanical signal to an electrical one. Second, the sense of hearing in both insects and vertebrates is characterized by highly nonlinear response functions, with power-law growth of the response to stimuli of increasing amplitude (8–11). Third, both systems reveal the presence of an energy-consuming amplification process. In certain species, this process can manifest itself in active limit cycles: Some vertebrates exhibit innate hair-bundle oscillations (12–14) and spontaneous otoacoustic emissions (15, 16), while male mosquitoes display naturally occurring self-sustained oscillations of their flagella (Fig. 1 *A* and *B*) (17, 18). Fruit flies possess similar machinery and can exhibit chemically induced self-sustained flagellar oscillations, though this active system naturally resides in a quiescent state (19).

Nonlinear dynamics models have provided an extensive theoretical framework for the study of vertebrate hearing. Specifically, theoretical models based on the normal form equation for the Hopf bifurcation have been shown to capture many of the key phenomena that characterize the auditory system (20, 21). The compressive nonlinearity of the response, amplification of low-intensity sound, frequency selectivity, and the presence of active, autonomous oscillations are all well described by this simple equation, showing consistency across many measurements performed on different species.

Important differences, however, arise between the nonlinear dynamics underlying insect hearing, compared to those established to hold for vertebrates. In all of the vertebrate systems studied, the frequency characterizing innate oscillation coincides with that of optimal sensitivity of detection. In insects, however, these frequencies can differ (18, 22–24). Male mosquitoes of several species have been shown to exhibit self-sustained oscillations (SSOs) of their flagella, with oscillation frequencies comparable to the wingbeat frequency of the corresponding females. As these SSOs have been shown to synchronize with female wingbeats, they are believed to serve as an amplification and filtering mechanism, enhancing the male’s ability to detect a female and thus improving his chances of mating. Surprisingly, however, the optimal tuning of the male’s sensory neurons does not coincide with the frequency of interest—female wingbeat frequency—or the male’s own SSO frequency (25, 26). Instead, the optimal frequency of detection of the sensory neurons was shown to coincide with a distortion product corresponding to a combination of the male’s own wingbeat with that of the female (Fig. 1*C*) (18, 22, 24).

Distortion products (DPs), also referred to as phantom tones, are a generic feature of nonlinear systems and are ubiquitous in all active amplifiers. They have previously been observed in vertebrates at the level of individual hair cells (27) as well as in vivo; upon presentation of two stimulus waves, these phantom tones are manifested as additional peaks in the power spectra, which correspond to linear combinations of the two applied frequencies (16, 28–30). As vertebrate systems are dominated by a cubic nonlinearity, the highest peaks in the distortion spectra have been shown to be third order. However, in vertebrates, these distortions are a byproduct of the amplification mechanism, whose main purpose is to enhance the detection of primary tones (PTs).

While PT detection has been extensively studied, to the best of our knowledge, no model has yet explored the effects of DP detection. In the current work, we use a generic model for the mosquito’s auditory system to show that detecting DPs of the stimulus, as opposed to the PTs, enhances the frequency selectivity and the speed of response. As frequency discrimination and temporal acuity are likely to be essential for reliable communication (31), we speculate that this mechanism is important for auditory detection by mosquitoes. We show the phenomenon to be ubiquitous, applicable across many orders of DPs, and to constitute a general design principle for sharpening the frequency selectivity of an active detector.

Detection poised at a DP, however, does include a significant trade-off, in that tuning is sharpened at the expense of sensitivity of detection. As insects must be capable of detecting faint signals, this limitation would prove harmful. We hence consider the active signal amplification provided by the self-sustained oscillations of the flagellum and determine to what extent it can compensate for the loss of sensitivity inherent to DP detection. Thus, we determine the effects of connecting several detectors in a cascade, in which the response of each oscillator provides the input into the next. This is a natural framework to describe the mechanical and electrical components of the mosquito’s auditory system, as the two elements have different tuning curves and can be regarded as two separate filters. We demonstrate that cascading the signal through several layers of detectors enhances amplification for weak and near-resonance stimuli, while providing greater attenuation of large-amplitude and off-resonance signals. The sensitivity enhancement improves with additional elements in the cascade, but with diminishing returns.

Finally, we combine the effects of DP detection and signal cascading in a configuration that mimics the male mosquito’s auditory system. We consider a two-oscillator cascade, consisting of a one oscillator tuned to the female wingbeat frequency, which feeds its response into a second oscillator tuned to the cubic DP frequency. We regard this two-oscillator system as a generic model, in which normal-form equations are used to explore and describe the basic features of the male mosquito’s auditory system. For instance, the two proposed oscillators may reflect, respectively, mechanical and electrical tuning in the system. The model contains only two free parameters, which control the dynamic state of the cascade. We evaluate the signal-detection capabilities of this composite system in terms of its sensitivity to weak stimuli, frequency selectivity for the female wingbeats, and the speed of response.

We explore the effects of the parameter choices on the performance of the system as a signal detector. Across all of the regimes studied, the full system exhibits an enhanced frequency selectivity, showing up to two orders of magnitude improvement compared to a single, PT detector. Furthermore, we show that this enhanced frequency selectivity does not come with the cost of a slow response, as would be the case for a sharply tuned linear oscillator. Rather, the system responds faster than a harmonic oscillator of equal quality factor, showing an order of magnitude improvement in the speed of response. These rapid, frequency-selective responses may be essential for the male mosquito to identify and locate the female using her transient, narrowband flight tones. While the model is inspired by recent experimental observations of the mosquito auditory system (18, 22, 24), the findings are generic. Tuning to the distortion products provides a way to enhance the frequency selectivity of a detector without compromising the speed of the response.

## Results

We first explore the full features of DP detection, by considering an individual Hopf oscillator tuned to one of the DPs of a two-tone stimulus. Second, we explore how recursively cascading the response of one oscillator into another affects signal detection to a single, near-resonance tone. Each active element in the cascade is represented by a normal form equation for the Hopf oscillator. Finally, we combine these two features to propose a model of the auditory system of the male mosquito. We consider the simplest case, in which there are only two oscillators in the cascade, and the second oscillator is tuned to a cubic distortion product of the male and female wingbeats. We evaluate the performance of the detectors by computing sensitivity to weak stimulus, frequency selectivity, and speed of response.

### Distortion-Product Tuned Detectors.

We first demonstrate the effects of tuning an active detector to one of the distortion product frequencies of a two-tone stimulus. We consider a system described by a complex variable, z(t), which is governed by the normal form equation for the supercritical Hopf bifurcation (32),

[1] $$\begin{array}{c} \frac{\mathrm{dz}}{\mathrm{dt}}=\left(\mu +i\omega_{0}\right)z-{\left|z\right|}^{2}z+F_{1}e^{i\omega_{1}t}+F_{2}e^{i\omega_{2}t}, \end{array}$$

where $F_{1}$ and $F_{2}$ are the stimulus amplitudes, $\omega_{1}$ and $\omega_{2}$ are the stimulus frequencies, and μ represents the control parameter of the system. For μ<0, the system resides in the quiescent regime, while for μ>0, the system displays self-sustained oscillations at the characteristic frequency, $\omega_{0}$. The Hopf bifurcation occurs at the critical point between these two regimes (μ=0). Without loss of generality, we let $\omega_{2}\geq \omega_{1}$.

**Fig. 2.**
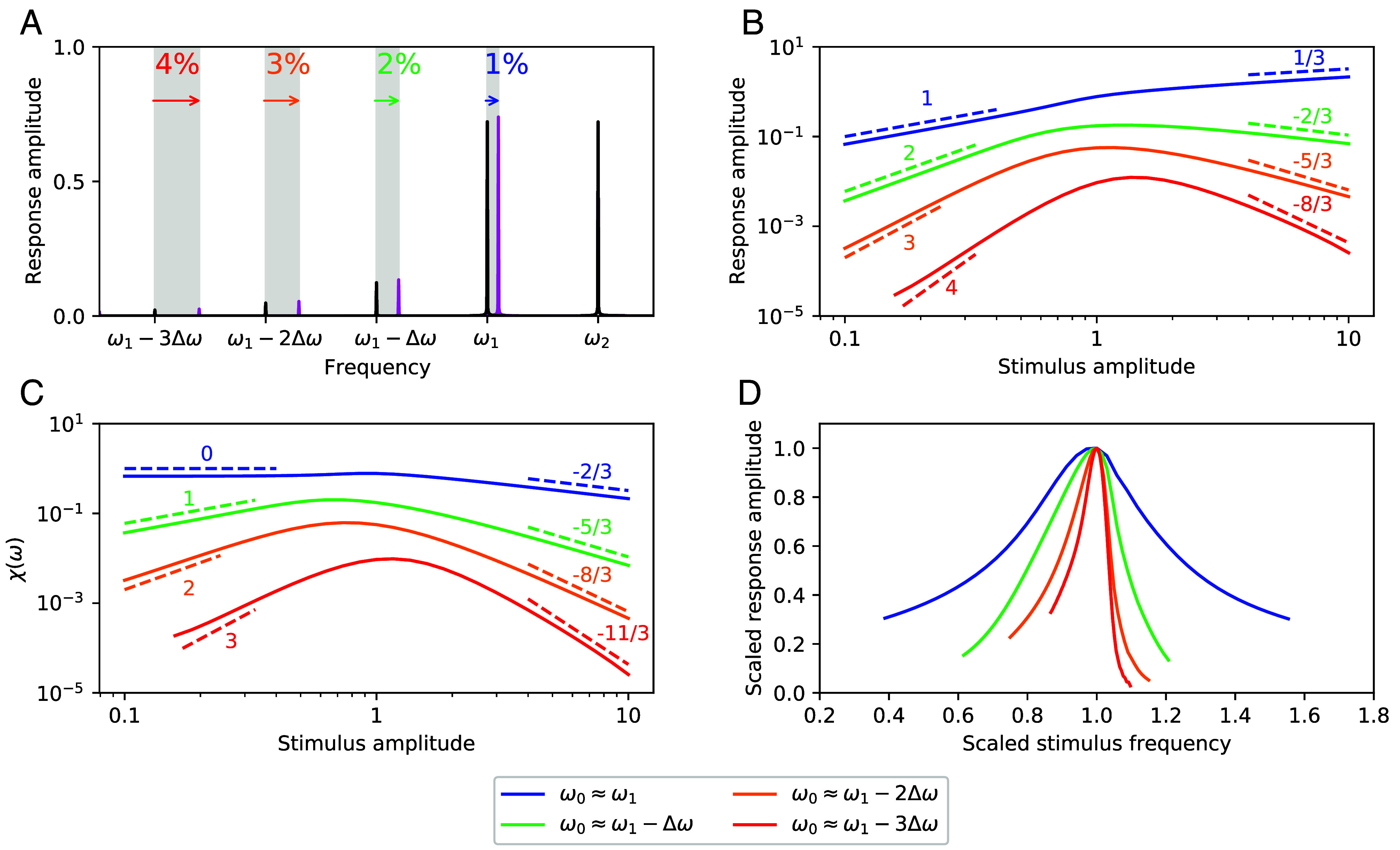
DP detection. (*A*) Fourier transform of the response of a Hopf oscillator to a two-tone stimulus (black curve). Response amplitudes fall off rapidly with increasing DP order. The arrows illustrate the frequency shifts associated with increasing $\omega_{1}$ by 1% (pink curve). Response amplitudes (*B*) and Sensitivity (*C*) of a single Hopf oscillator exposed to a range of stimulus amplitudes, $F_{1}$ (*Materials and Methods*). Various power-law relationships predicted by our analytic calculations (*SI Appendix*) are labeled and plotted with dashed lines. (*D*) Scaled response amplitude to a frequency sweep in $\omega_{1}$. For panels (*A*–*C*), $F_{2}=1$ and $\frac{\omega_{2}}{\omega_{1}}=1.1$. For panel (*D*), $\omega_{1}$ is modulated around 2π, while $\omega_{2}=1.1\times 2\pi$, and $F_{1}=F_{2}=0.1$. For all panels, μ=−0.1.

This system exhibits nonlinear responses at the distortion product frequencies,

[2] $$\begin{array}{c} \omega_{p,q}=p\omega_{1}-q\omega_{2}, \end{array}$$

where p and q are integers, and the distortion product order is defined as the sum of the magnitudes (|p|+|q|). The cubic term in Eq. **1** results in distortion products of only odd order. The distortion products lower than $\omega_{1}$ can be represented as $\omega_{p,q}=\omega_{1}-\frac{1}{2}\left(|p|+|q|-1\right)\Delta \omega$, where $\Delta \omega =\omega_{2}-\omega_{1}>0$. It has been demonstrated that distortion-product amplitudes fall off exponentially with increasing order, provided that the frequency difference between the two tones is much greater than the active bandwidth of the oscillator (33). In Fig. 2*A*, we illustrate this effect. The figure further demonstrates that the sensitivity to modulations in the stimulus frequency, $\omega_{1}$, increases with increasing distortion-product order. We propose that this effect could serve to enhance frequency selectivity and frequency discrimination of external tones. This can be understood, in part, through calculating the changes in distortion-product frequency with respect to $\omega_{1}$,

[3] $$\begin{array}{c} \frac{d\omega_{p,q}}{d\omega_{1}}=p, \end{array}$$

which increases with increasing DP order. Note that we are considering $\omega_{1}$ to be the stimulus tone of interest, while examining the low-frequency distortion products. However, these effects can also be seen for modulation in $\omega_{2}$ and for high-frequency distortion products.

We chose two stimulus tones that differ minimally in frequency, $\frac{\omega_{2}}{\omega_{1}}=1.1$, so as to observe several orders of distortion products. We calculate the phase-locked response amplitude and the level function of sensitivity, χ(ω) (*Materials and Methods*), over a range of stimulus amplitudes, $F_{1}$. For all stimulus magnitudes, we find that the amplitude of the response and the sensitivity decrease with increasing DP order (Fig. 2 *B* and *C*). The oscillator tuned to the primary tone exhibits the expected linear growth for small stimulus amplitudes and compressive response with 1/3 power law for large stimulus amplitudes (34).

However, when the detector is tuned to a distortion product, the response to weak signals shows nonlinear growth, following integer power laws, with more rapid growth for higher DP orders. For strong signals, the response of the DP-tuned detectors, surprisingly, decreases with increasing stimulus amplitude. The decline in the response likewise follows power laws, with more rapid falloff observed for increasing DP order (Fig. 2*B*). The power laws in both the weak and strong stimulus regimes are in good agreement with our analytic calculations (*SI Appendix*). Between these two regimes, the responses of the DP-tuned detectors display maxima when the two stimulus tones have approximately equal intensity. The level function of sensitivity shows similar behavior, but with an additional factor of $\frac{1}{F_{1}}$, and thus a reduction in the power laws.

The enhanced growth at weak stimulus levels could enable the detector to resolve variations in the volume of faint sounds, while the extreme compression at higher amplitudes could maintain a large dynamic range. This alternative detection scheme hence exhibits advantages over the traditional response function of an oscillator tuned to the primary tone, but it entails a reduction of the overall sensitivity. Further, the nonmonotonic nature of the response curves implies an ambiguity in identifying stimulus levels, as there is not a one-to-one relationship of input and output amplitudes. This ambiguity at the level of an individual oscillator could, however, be resolved in an array of such detectors.

The most striking benefit of tuning a detector to a distortion product emerges when calculating the frequency selectivity of the response. Notably, the quality factor of the response increases when the system is tuned to a distortion product (*SI Appendix* for an analytic calculation). The frequency selectivity continues to increase with increasing DP orders (Fig. 2*D*). This feature arises as a result of several effects. First, modulations in the primary tones are magnified at the DP level (Eq. **3**). This magnification, in turn, leads to a more narrow range of frequencies over which the detector responds. Second, large stimulus amplitudes that would normally saturate the response to primary tones are attenuated at the DP level, due to the previously described compression. These effects result in sharper tuning curves and a larger quality factor.

### Cascade of Nonlinear Amplifiers.

**Fig. 3.**
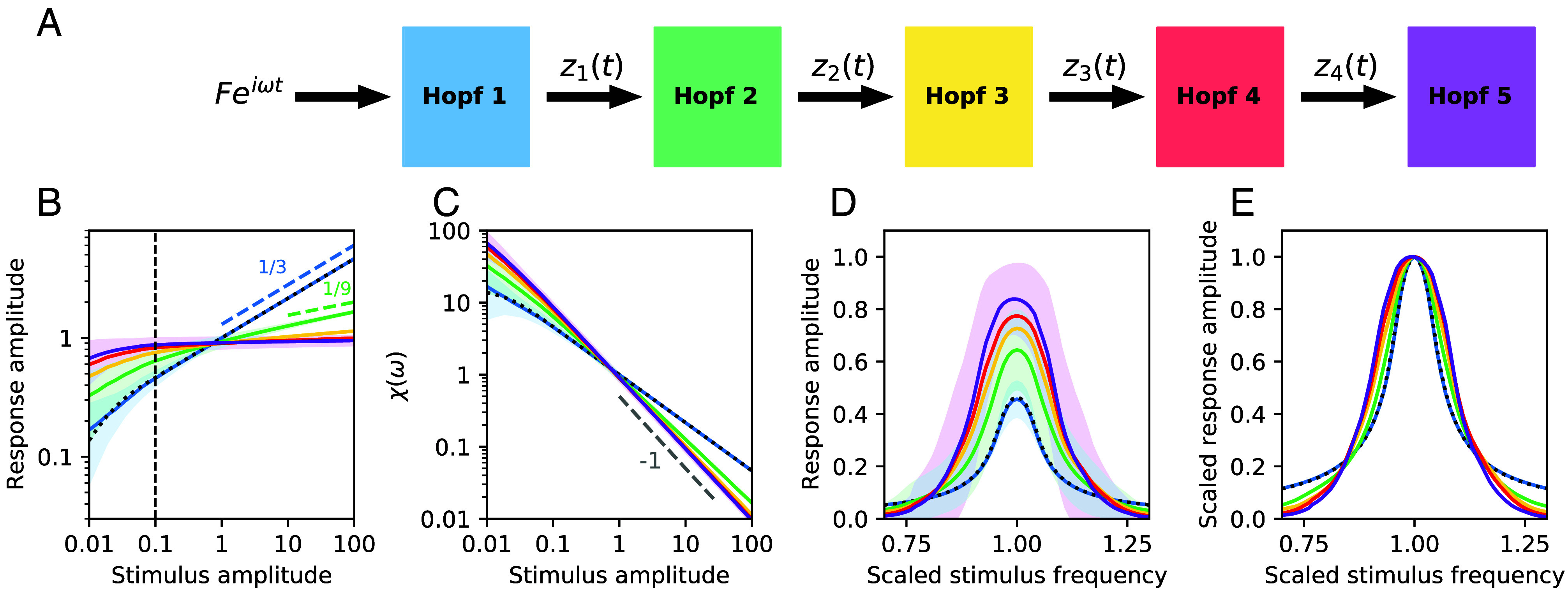
Amplifier cascade. (*A*) Schematic of the external signal passing through five layers of detection. (*B*) Response amplitudes at the mean characteristic frequency for a range of stimulus amplitudes. In the strong-stimulus regime, 1/3 and 1/9 power-law growth are shown with dashed lines. (*C*) Corresponding level functions of sensitivity for the response amplitudes in (*B*). In the strong-stimulus regime, the level functions of sensitivity approach a $\frac{1}{F}$ dependence (gray, dashed line) with increasing number of oscillators. (*D*) Response amplitude for a range of stimulus frequencies (scaled by the mean characteristic frequency) with F=0.1, as indicated by the vertical dashed line in (*B*). (*E*) Same as (*D*) but with curves normalized to their peak values. The colors of the curves correspond to the layer of cascading, as illustrated in (*A*). All $\mu_{j}$ values were chosen pseudorandomly from a Gaussian distribution with mean 0 and SD 0.1. All $\omega_{j}$ values were, likewise, chosen from a Gaussian distribution with mean 2π and SD 0.1×2π. The solid curves and shaded regions correspond to the mean and SD of each measure obtained from 100 unique parameter combinations. For comparison, the black, dotted curves correspond to a single Hopf oscillator tuned precisely to the stimulus frequency ($\omega_{1}=2\pi$) and poised at the bifurcation point, ($\mu_{1}=0$).

Next, we establish the effects of recursively cascading the response of one Hopf oscillator as input into another (Fig. 3*A*). This configuration is motivated by the multiple components of the mosquito’s auditory system, where we regard the flagellum and the mean field of the neural elements as two distinct oscillators, with different characteristic frequencies and control parameters. We note that this configuration can be generalized to describe other systems that exhibit multiple components. For example, signal detection by the hair cells of vertebrates involves the mechanical deflections of the hair bundles, which pivot in response to sound and vibration. These mechanical receptors modulate the opening probability of the transduction channels embedded in the hair bundle and cause an influx of ionic current into the cell body (35, 36). The hair-cell soma acts as an electrical filter, and in some species, an electrical resonator (15). The signal is then propagated to the neuron connected to the base of the soma. Thus, this system is composed of multiple distinct layers and could likewise be modeled with a cascade of oscillators.

To explore these effects independently of those of DP detection, we apply a single-tone, near-resonance stimulus to the system and measure the phase-locked response. The dynamics of the first oscillator are described by

[4] $$\begin{array}{c} \frac{dz_{1}}{\mathrm{dt}}=\left(\mu_{1}+i\omega_{1}\right)z_{1}-{\left|z_{1}\right|}^{2}z_{1}+Fe^{i\omega t} \end{array}$$

and the dynamics of the following elements in the cascade are described by

[5] $$\begin{array}{c} \frac{dz_{j}}{\mathrm{dt}}=\left(\mu_{j}+i\omega_{j}\right)z_{j}-{\left|z_{j}\right|}^{2}z_{j}+z_{j-1}\left(t\right), \end{array}$$

where the indexing starts at j=2 and terminates at $j=j_{\max}$. With identical parameters, it is well understood that amplification is enhanced with increasing number of amplifiers (37, 38). However, to test for robustness of the cascading scheme, we allow the control parameters to deviate from the bifurcation point. Further, we allow the characteristic frequencies of the oscillators to deviate from each other. Prior to simulating the dynamics, each $\mu_{j}$ is chosen pseudorandomly from a Gaussian distribution centered at 0, with a SD of 0.1. Likewise, each $\omega_{j}$ is chosen from a Gaussian distribution centered at 2π, with a SD of 0.1×2π.

Despite the imprecise tuning of the parameters, cascading a signal through multiple elements enhances several of the features exhibited by individual Hopf oscillators. At low stimulus amplitudes and in the vicinity of the mean characteristic frequency, the nested oscillators exhibit higher amplification of the signal, increasing the response and the sensitivity (Fig. 3 *B* and *C*). Further, the system exhibits stronger compression of large-amplitude stimuli. When a single Hopf oscillator receives a large-amplitude stimulus, the response grows as $F^{\frac{1}{3}}$ due to the cubic term. For a cascading system receiving a strong stimulus, the response of the jth element grows as $F^{{\left(\frac{1}{3}\right)}^{j}}$. After several layers of cascading, the response flattens out and approaches a constant amplitude (Fig. 3*B*), and the sensitivity approaches a $\frac{1}{F}$ dependence (Fig. 3*C*).

Further, we find that the cascade of Hopf oscillators amplifies only near-resonance stimuli, while attenuating the response at off-resonance frequencies (Fig. 3 *D* and *E*). Thus, the narrow range of frequencies in the immediate vicinity of the mean characteristic frequency is enhanced, with a sharp decrease and vanishing tail as frequency is varied. The frequency selectivity of the system is therefore slightly enhanced in comparison to that of an individual element. However, the effect is not as prominent as the enhanced frequency selectivity that arises from DP detection (Fig. 2*D*).

We note that the benefits of the proposed cascade of Hopf oscillators exhibit different features than what is observed by simply tuning one oscillator precisely to the stimulus frequency and poised precisely at the bifurcation point (dotted curves in Fig. 3). In Fig. 3*B*–*E*, we compare the sensitivity and frequency selectivity of the response. As can be seen, the enhancement of sensitivity to weak signals, attenuation of strong signals, and frequency selectivity of the response is improved as more layers are added to the cascade (increasing $j_{\max}$). Most of the enhancement, however, already occurs in the first several layers, with diminishing returns upon continuing the cascade. Further, the benefits are robust to imperfect tuning of the system parameters.

### Generic Model of the Mosquito’s Auditory System.

Finally, we combine the two effects described previously in order to build a simple model for signal detection by male mosquitoes. The feather-like flagellum at the end of the antenna of a mosquito oscillates in response to an incoming sound wave. The base of the antenna contains the Johnston’s organ, which has a bowl-like structure with rotational symmetry. The pivots of the antenna modulate the open probability of the transduction channels embedded in the ciliated sensory neurons that connect to the antennal base (Fig. 1 *A* and *B*). To construct a simplified model of these elegant structures, we describe the flagellar position by a time-dependent complex variable, $z_{1}\left(t\right)$. For simplicity, we describe the mean field of the active neuronal elements by a second complex variable, $z_{2}\left(t\right)$. Both of these state variables are governed by the normal form equation for the supercritical Hopf bifurcation,

[6] $$\begin{array}{c} \frac{dz_{1}}{\mathrm{dt}}=\left(\mu_{1}+i\omega_{1}\right)z_{1}-{\left|z_{1}\right|}^{2}z_{1}+F_{f}e^{i\omega_{f}t}+F_{m}e^{i\omega_{m}t} \end{array}$$

[7] $$\begin{array}{c} \frac{dz_{2}}{\mathrm{dt}}=\left[\mu_{2}+i\left(2\omega_{1}-\omega_{m}\right)\right]z_{2}-{\left|z_{2}\right|}^{2}z_{2}+z_{1}\left(t\right), \end{array}$$

where the sinusoidal forcing terms represent the input stimuli, with $F_{f}$ and $F_{m}$ denoting the amplitudes and $\omega_{f}$ and $\omega_{m}$ the frequencies of the female and male wingbeats, respectively. The male flagellum exhibits a characteristic frequency $\omega_{1}$, while $\mu_{1}$ represents the control parameter of the flagellum. For $\mu_{1}<0$, the flagellum is in the quiescent state, while for $\mu_{1}>0$, the flagellum exhibits self-sustained oscillations powered by an internal energy source. This control parameter, $\mu_{1}$, thus determines the dynamic state of the flagellum. The dynamic state of the neural mean field is, likewise, controlled by $\mu_{2}$. We consider all four permutations of the control parameters ($\mu_{1}=\pm 0.1$ and $\mu_{2}=\pm 0.1$), so as to explore all possible dynamic states of the full system.

**Fig. 4.**
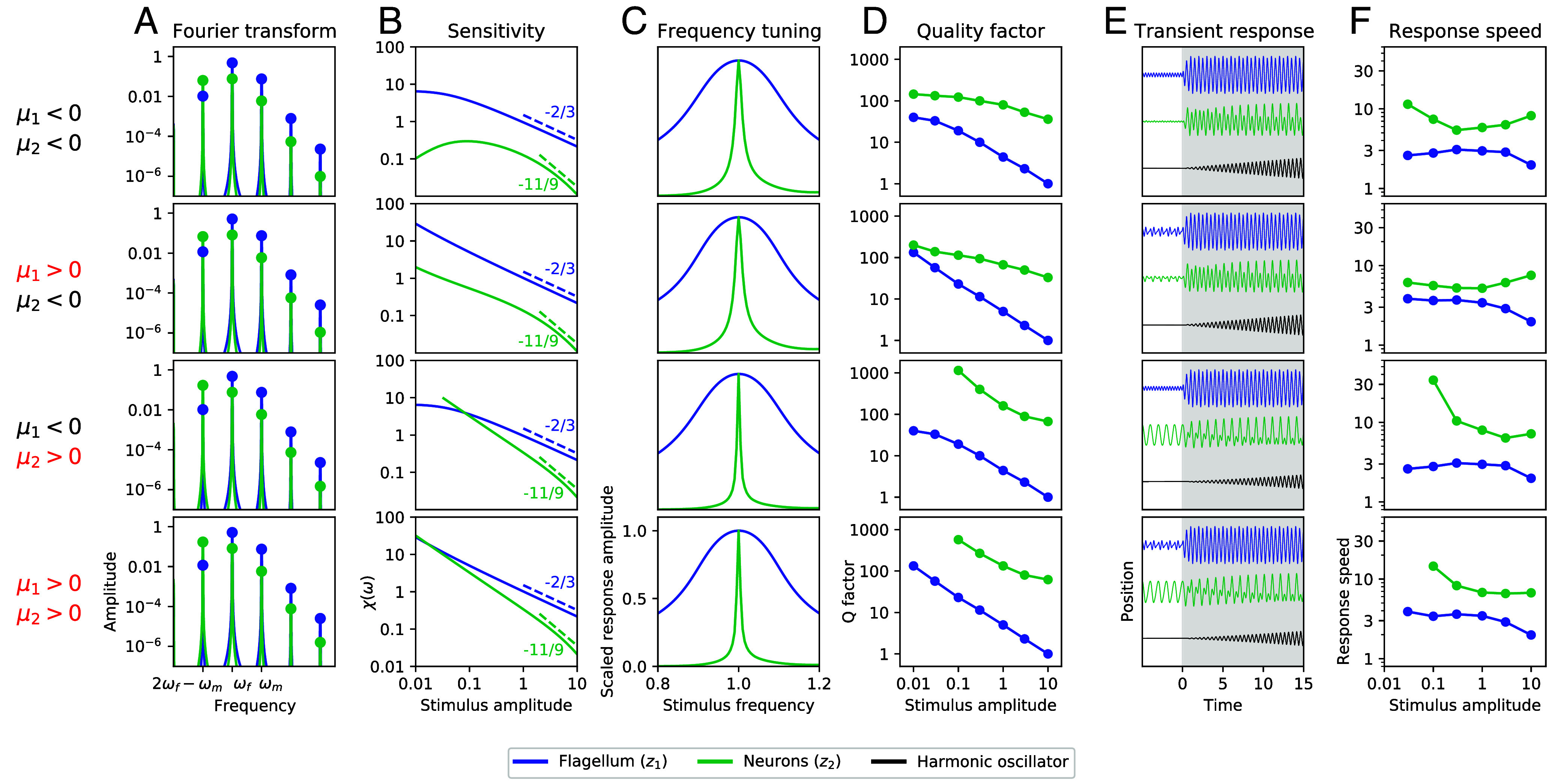
Mosquito model. Responses of the first (blue) and second (green) oscillators in the composite system, for all four permutations of control parameters ($\mu_{1}=\pm 0.1$ and $\mu_{2}=\pm 0.1$). (*A*) Fourier transforms of the responses with $\omega_{f}=\omega_{1}$ and $F_{f}=F_{m}=1$. (*B*) Level functions of sensitivity for a range of stimulus levels, $F_{f}$ (*Materials and Methods*). Dashed lines indicate the strong-stimulus power-law relationships predicted by analytic calculations (*SI Appendix*) (*C*) Tuning curves scaled to the frequencies at peak response and normalized to the peak amplitudes. (*D*) Quality factors for a range of stimulus levels, $F_{f}$. (*E*) Transient response to the abrupt onset of a female flight tone (gray shaded region) with $\omega_{f}=\omega_{1}$ and $F_{f}=3$. For comparison, responses of simple harmonic oscillators with quality factors equal to that of the neural mean field (second oscillator) are plotted in black. Note that the male’s flight tone is present for the entire simulation. (*F*) Response speeds for a range of stimulus levels, $F_{f}$. For all panels, $F_{m}=1$, $\omega_{1}=2\times 2\pi$, and $\omega_{m}=3\times 2\pi$.

Although several orders of distortion products may be important for signal detection, we consider only the simplest case, where the neuronal elements are tuned to the cubic distortion product between the characteristic frequency of the flagellum and the male’s wingbeat frequency, $2\omega_{1}-\omega_{m}$. We use experimental data obtained from prior studies on several mosquito species to approximate the relationship between the frequencies (39). Hence, we consider a stimulus frequency ratio of $\frac{\omega_{m}}{\omega_{f}}\approx 1.5$, and set the male flagellum to be tuned near the female wingbeat frequency $\omega_{1}\approx \omega_{f}$, which makes the neuronal elements tuned near the cubic distortion product frequency, $2\omega_{1}-\omega_{m}\approx 2\omega_{f}-\omega_{m}$ (Eq. **5**,**7**), consistent with the experimental results. We show the response of this system in frequency space for all four control parameter permutations (Fig. 4*A*). We find that the largest peaks occur at the two primary tones and the cubic distortion product ($2\omega_{f}-\omega_{m}$). The response of the second oscillator at this DP frequency is particularly large, often exceeding its response at the primary tones.

We calculate the sensitivity of both oscillators to a female flight tone (Fig. 4*B*; *Materials and Methods*). Our definition of sensitivity is based on the amplitude of the response. However, detection can also occur through phase entrainment (*SI Appendix*, Fig. S1). As predicted in the prior sections, for $\mu_{1}=\mu_{2}<0$, the second oscillator is approximately two orders of magnitude less sensitive to weak signals than the first oscillator. However, when autonomous oscillations are introduced to the first oscillator ($\mu_{1}>0$), the difference in sensitivity reduces to one order of magnitude. When both oscillators are poised in the oscillatory regime, the sensitivity to weak stimulus is fully recovered. Further, the high attenuation of strong signals is preserved in the second oscillator. Most strikingly, we find that the tuning curves are significantly sharper for all four control parameter permutations (Fig. 4*C*). We quantify this effect by computing the quality factors of the tuning curves. In Fig. 4*D*, we show how the quality factor varies with stimulus level, $F_{f}$, for each of the two oscillators. For all four control parameter choices and all stimulus levels, we find that the second oscillator is more frequency selective than the first, often by a factor of 10 to 100. As discussed previously, this improvement in frequency selectivity is a consequence of DP tuning.

Last, we show the transient response to the abrupt onset of the female flight tone (Fig. 4*E*), in order to test whether the enhanced frequency selectivity has an adverse effect on the speed of the response. In all cases, we find that the flagellum reaches steady state after 1 to 3 oscillations, while the neural mean field has a transient lasting about 10 cycles. For comparison, we plot the transient responses of harmonic oscillators with quality factors equal to those of the neuronal mean fields. These linear systems show about 100 oscillations before reaching steady state. To quantify this effect, we measure the speed of the transient response induced by an abrupt stimulus onset and compare this to the response of a harmonic oscillator of equal quality factor (*SI Appendix*). A response speed of 10 indicates that the system reaches steady state 10 times faster than a harmonic oscillator of equal quality factor. We find that the response speed of the second oscillator is consistently higher than that of the first, for all stimulus levels (Fig. 4*F*). We find that, regardless of the dynamic state of the system, DP detectors can be more than 10 times as frequency selective as the corresponding PT detectors, while still maintaining a response speed about 10 times faster than a linear system of equal quality factor.

## Discussion

Prior numerical models of mosquito hearing have accounted for several of the experimentally measured phenomena of the mosquito’s auditory system, including self-sustained oscillations of the flagellum, the nonlinear and hysteretic response function that results from stimulus amplitude ramps, and the appearance of a frequency-doubling component at the neuronal level. These phenomena were described using a microscopic integrate-and-twitch model (40) that tracks the dynamics of the individual neuronal elements, which in turn apply impulsive forcing back onto the flagellum. This model has been further extended to the continuum limit (41). A phenomenological model has also been developed, in which the flagellum is assumed to be a simple harmonic oscillator, while the active neuronal units are represented either by a single Hopf oscillator representing the mean-field neural response (42), or an array of coupled Hopf oscillators, each representing a cluster of active neural elements (43).

With the current study, we constructed a general model for mosquito hearing, based on dynamical systems theory, aimed to capture the basic features of this remarkable auditory system. While the normal form equation for the Hopf bifurcation, tuned for optimal detection of a primary tone, has been shown to describe all the key characteristics of the vertebrate system, it does not readily generalize to that of insects. We aimed here to develop a framework, which can then be fine-tuned to describe specific behaviors of different mosquito species, and possibly those of other flagellar insects. We propose that two key features emerge in constructing a general model: cascading of nonlinear amplifying elements and detection of distortion products.

Highly nonlinear responses have been measured in the antennae of the fruit fly (44), honey bee, and several species of mosquito (11). These systems exhibit compressive nonlinearities at large stimulus amplitudes (45), showing nontrivial power-law relationships. For example, the fruit fly exhibits a 2/3 power-law growth in the mechanical response of its receiver (9), while exhibiting a compressive, and even nonmonotonic neural response (44). We speculate that mechanisms similar to distortion product detection may be uncovered in other insect species, as we observe nontrivial power-law growth and nonmonotonic response functions in our model (Fig. 2 *B* and *C*). We also note that a highly compressive neural response has been measured in the mosquito auditory system, with the electrical response growing only about an order of magnitude over the span of three orders of magnitude in stimulus amplitude (45). This compressive response is consistent with a 1/3 power law, which can be observed in our numerical model when the second oscillator resides in the quiescent state (*SI Appendix*, Fig. S1*C*).

The most noteworthy difference between PT and DP detection lies in the frequency selectivity of the response. DP detectors display much sharper frequency tuning, with quality factors increasing with increasing DP order. The degree to which the quality factor is improved for a cubic DP detector increases and diverges with weaker stimulus amplitudes (*SI Appendix*). We note that the sharpening of the response curve occurs regardless of the dynamic state of the system. This enhancement is a consequence of two mechanisms. First, the DP detector attenuates any incoming, off-resonance signals, narrowing the range in frequencies over which detection will occur. Second, DP frequencies are sensitive to modulations in the PT frequency, with sensitivity growing with increasing DP order. This results in a further narrowing of this detection range.

A single, deterministic Hopf oscillator acting as a PT detector can also exhibit high frequency selectivity, with the quality factor of the response diverging as the forcing amplitude goes to zero (*SI Appendix*). However, when stochastic fluctuations are introduced, the frequency selectivity quickly deteriorates, especially if the system is poised near the Hopf bifurcation (46). We speculate that a single Hopf oscillator poised near criticality would be insufficient for detecting and isolating the transient, narrowband signals produced by the female’s wingbeats, as mosquitoes and other insects often reside in noisy swarm environments (31). The configuration of detectors we propose provides an alternative mechanism for achieving high frequency selectivity, while avoiding a slow response time. Our analytic calculations demonstrate that DP detectors, in general, produce high quality factors, which diverge more rapidly than those of PT detectors for vanishing forcing amplitudes (*SI Appendix*).

Inspired by the auditory system of the mosquito, we explored the effects of connecting several Hopf oscillators in a cascade, with the response of each oscillator acting as the stimulus for the next. We note that this detection scheme differs from arrays of active Hopf oscillators arranged tonotopically, which have previously been used to model the mammalian cochlea (47). The configuration used in this study is an attempt to mimic the distinct tuning curves measured in the mechanical and electrical components of the mosquito’s auditory system. We conceptualize these two components as two distinct oscillators with mismatching tuning curves, rather than a single oscillator with multiple resonance peaks. There may be additional levels of cascading at the neuronal level, which could be readily accounted for by additional elements in the cascade. We found that cascading enhances sensitivity to weak stimuli, while more significantly compressing the response to large-amplitude signals. Further, each level of detection distorts the shape of the response curve, making the system more sensitive to near-resonance stimulus and less sensitive to off-resonance stimulus.

Last, we constructed a system that exhibits both of the described effects by feeding the response of a PT detector into a DP detector. We chose characteristic frequencies to reflect those observed in experimental measurements of mosquitoes. This generic, two-oscillator signal detector exhibits high sensitivity to weak stimuli, nonlinear compression of strong stimuli, sharp tuning curves, and a rapid response. We conceptualize the mosquito’s auditory system as a mechanical resonator feeding its response into an electrical oscillator of different characteristic frequency. Intuitively, this mismatch in frequency tuning should attenuate inputs of any frequency and yield poor performance as a signal detector. However, using this generic model for auditory detection and considering the nonlinear distortion products of the response, we motivate this counterintuitive detection scheme. We show that this class of systems responds sensitively to weak stimulus, while attenuating strong signals. Most strikingly, this scheme yields immense frequency selectivity, which may be essential for the male mosquito to isolate the flight tone of a female within a noisy swarm environment.

Future theoretical work will entail introducing filtered noise into the system to account for the sound produced by the swarm environment. The frequency dispersion of the active neural elements should also be considered, to test the robustness of the model. Experimental data show that the SSOs of the male mosquito have quality factors of about 20 (18). However, the tuning curves in response to acoustic stimulus have a reduced quality factor (48), which, given the nonlinearity of the system, should reduce further with stronger stimulus. One challenge in developing a numerical model of this system is estimating the intensity of the male’s own wingbeats in comparison to the male’s minimum detection threshold of the female (49, 50). In the current work, we therefore considered a range of three orders of magnitude in this ratio, $F_{f}/F_{m}$, while ensuring that the flagellum does not entrain to the male’s own wingbeat, as supported by experimental data (18). Another limitation to our proposed model is that it does not capture the difference tone, $\omega_{m}-\omega_{f}$, which has been shown to be of importance in addition to the cubic DP (18, 45). We note that the quadratic and cubic DPs coincide when $\frac{\omega_{m}}{\omega_{f}}=1.5$, a ratio often measured in the flight tones of mosquitoes and the ratio used in this study. Future studies will elaborate on the current model and explore the relative importance of additional nonlinearities.

Future experiments should also entail measuring the power-law relationship between two-tone acoustic stimulus intensity and neuronal response amplitude for the various distortion products. To further test our theoretical predictions, the quality factors of these responses should also be measured and compared to the predicted signal-analytical gains. Here, especially the roles and molecular nature of the elusive control parameter, μ, will be of interest. It is tempting to speculate that it is linked to the reported vast efferent neuromodulation of the mosquito ear (48, 51). Intriguingly, both distortion products and efferent innervation are poorly understood features of human hearing, too.

## Materials and Methods

Numerical simulations were performed using the fourth order Runge–Kutta method with time steps of $10^{-3}$ and approximately $10^{5}$ time steps to ensure high frequency resolution.

### Response Amplitude and Sensitivity.

The Fourier transform of complex variable, z(t), takes the form,

[8] $$\begin{array}{c} \tilde{z}\left(\omega \right)=\frac{1}{T}\int_{-\frac{T}{2}}^{\frac{T}{2}}e^{-i\omega t}z\left(t\right)dt, \end{array}$$

where T is the observation time. We define the response amplitude as the magnitude of the Fourier component at the frequency of interest. For primary-tone detectors, this is computed by taking $|\tilde{z}\left(\omega_{1}\right)|$, where $\omega_{1}$ is the stimulus frequency of interest. However, for distortion-product detectors, we compute the response at the distortion-product frequency, $|\tilde{z}\left(\omega_{p,q}\right)|$, where $\omega_{p,q}$ is defined in Eq. **2**. To characterize the responsiveness at a particular stimulus amplitude, we calculate the level function of sensitivity,

[9] $$\begin{array}{c} \chi \left(\omega \right)=\frac{|\tilde{z}\left(\omega \right)|}{F_{1}}, \end{array}$$

where $F_{1}$ is the amplitude of the external stimulus of interest and $\tilde{z}\left(\omega \right)$ is computed using Eq. **8**.

## Supplementary Material

Appendix 01 (PDF)

## Data Availability

Custom code/scripts used in this study have been deposited in GitHub (https://github.com/jfaber3/Fig-Files-A-mosquito-inspired-theoretical-framework) (52).
