# Supplementary material for: A mosquito-inspired theoretical framework for acoustic signal detection
Source: Proc Natl Acad Sci U S A. 2025 Sep 5;122(36):e2500938122. doi: 10.1073/pnas.2500938122 (PMC12435297; doi:10.1073/pnas.2500938122)
Supplement: Supplementary file 1 — Appendix 01 (PDF) [file pnas.2500938122.sapp.pdf]

Supporting Information for

A Mosquito-Inspired Theoretical Framework for Acoustic Signal Detection

Justin Faber, Alexandros C Alampounti, Marcos Georgiades, Joerg T Albert and Dolores Bozovic

Justin Faber.
E-mail: faber@physics.ucla.edu

This PDF file includes:

Supporting text
Fig. S1
SI References

Supporting Information Text

Quality factor of primary-tone response. Here we estimate the quality factor of the response of a Hopf oscillator to a single-tone stimulus. We use

$$\frac{dz}{dt} = (\mu + i\omega_0)z - |z|^2 z + F(t), \tag{1}$$

where $F(t) = Fe^{i\omega t+\psi}$ is the stimulus with constant phase offset, ψ. We assume a response at the stimulus frequency of the form

$$z(t) = Re^{i(\omega t+\phi)}. \tag{2}$$

Inserting this ansatz into Eq. 1 and multiplying each side by its complex conjugate results in a cubic equation for the response amplitude,

$$F^2 = (R^3 - \mu R)^2 + (\omega - \omega_0)^2 R^2. \tag{3}$$

The response amplitude grows as $R \approx F^{\frac{1}{3}}$ for stimuli close to the characteristic frequency and for strong stimuli. However, for stimuli far from the characteristic frequency, the response can be approximated as $R \approx \frac{F}{|\omega-\omega_0|}$ if μ is small. We approximate the response near resonance up to second order in $(\omega - \omega_0)$, which gives the response amplitude as a function of the frequency detuning,

$$R \approx F^{\frac{1}{3}} - \frac{(\omega - \omega_0)^2}{6F}, \tag{4}$$

for systems close to the bifurcation ($|\mu| \ll 1$). This parabola can be used to estimate the width of the tuning curve at half its maximum in energy. The characteristic frequency divided by this width defines the quality factor of the response. For a single-tone stimulus, the approximation of the quality factor yields

$$Q_{\text{PT}} = \frac{1}{\sqrt{24(1-\frac{1}{\sqrt{2}})}}\left(\frac{\omega_0}{F^{2/3}}\right). \tag{5}$$

Quality factor of distortion-product response. We now approximate the quality factor of a Hopf oscillator tuned near the cubic distortion-product frequency ($\omega_0 \approx \omega_{dp} = 2\omega_f - \omega_m$). The forcing term takes the form $F(t) = F_f e^{i(\omega_f t+\psi_f)} + F_m e^{i(\omega_m t+\psi_m)}$. We use the frequency ratio described in the main text $\frac{\omega_m}{\omega_f} \approx 1.5$ and assume a response of the form

$$z(t) = R_f e^{i(\omega_f t+\phi_f)} + R_m e^{i(\omega_m t+\phi_m)} + R_{dp} e^{i(\omega_{dp} t+\phi_{dp})}, \tag{6}$$

where we are considering only the three largest frequency contributions. Inserting this into Eq. 1 and separating terms according to their frequency contribution gives rise to three equations for the three amplitudes.

Responses at the primary tones can be easily estimated from the equations corresponding to their respective frequency components. Since both tones are far from the resonance frequency, the responses can be estimated as

$$R_f \approx \frac{F_f}{|\omega_f - \omega_0|} \text{ and } R_m \approx \frac{F_m}{|\omega_m - \omega_0|}, \tag{7}$$

provided that the forcing is sufficiently weak. Isolating contributions to the distortion-product frequency gives

$$i\omega_{dp} R_{dp} = (\mu + i\omega_0)R_{dp} - R_{dp}(2R_f^2 + 2R_m^2 + R_{dp}^2) + R_f^2 R_m e^{(2\phi_f - \phi_m - \phi_{dp})}. \tag{8}$$

Computing the magnitude of this relationship yields

$$R_f^4 R_m^2 = (\delta\omega R_{dp})^2 + (R_{dp}^3 + 2R_f^2 R_{dp} + 2R_m^2 R_{dp} - \mu R_{dp})^2, \tag{9}$$

where $\delta\omega = \omega_{dp} - \omega_0 = 2\omega_f - \omega_m - \omega_0$. Note that modulations in ω_f are magnified by a factor of 2 in the distortion product. This equation for R_{dp} can be written in the same form as the single-tone case,

$$F_{\text{eff}}^2 = (R_{dp}^3 - \mu_{\text{eff}} R_{dp})^2 + (\delta\omega)^2 R_{dp}^2, \tag{10}$$

where $F_{\text{eff}} = R_f^2 R_m$ and $\mu_{\text{eff}} = \mu - 2(R_f^2 + R_m^2)$ are the effective forcing and effective control parameter, respectively (1). Notice that the effective forcing grows as F_f^2. This explains the quadratic growth in the distortion-product response seen in Fig. 2B.

Since this equation for R_{dp} has the same form as the single-tone relationship, we can use the same second-order expansion as described for the single-tone case if this system resides near the effective bifurcation point ($|\mu_{\text{eff}}| \ll 1$). We compute the quality factor with respect to variations in the distortion-product frequency,

$$Q_{\text{DP}} = \frac{1}{\sqrt{24(1-\frac{1}{\sqrt{2}})}} \left(\frac{\omega_0(\omega_m - \omega_f)^2}{(\frac{1}{2}F_f^2 F_m)^{2/3}} \right). \quad [11]$$

When comparing Q_{DP} to Q_{PT}, a factor of 2 is gained because modulations in $\delta\omega$ correspond to twice those in the female stimulus frequency. However, this factor of 2 cancels with the factor of 2 loss from the distortion-product detector being tuned to half the frequency of the primary tone. Taking the female tone to be the frequency of interest in the single-tone case, we can estimate by how much the quality factor is improved from detecting the first cubic distortion product instead of the primary tone,

$$\frac{Q_{\text{DP}}}{Q_{\text{PT}}} = \frac{(\omega_m - \omega_f)^2}{(\frac{1}{2}F_f F_m)^{2/3}}, \quad [12]$$

which continues to grow for weaker stimulus levels.

Power-law growth of distortion-product response. We now estimate the distortion-product growth rates in the limits of very weak and very strong stimulus. Again, we consider the two-tone stimulus applied to the Hopf oscillator,

$$\frac{dz}{dt} = (\mu + i\omega_0)z - |z|^2 z + F_1 e^{i\omega_1 t} + F_2 e^{i(\omega_1 + \Delta\omega)t}, \quad [13]$$

where the system is tuned near the Nth distortion product ($\omega_0 \approx \omega_1 - N\Delta\omega$). We assume a response of the form

$$z(t) = R_1 e^{i(\omega_1 t + \psi_1)} + R_2 e^{i([\omega_1 + \Delta\omega]t + \psi_2)} + \sum_{n=1}^{N} A_n e^{i([\omega_1 - n\Delta\omega]t + \phi_n)}. \quad [14]$$

Note that we are considering the distortion products only on one side of the primary tones. The others are far outside the tuning range of the system and contribute minimally to the solution. It has been shown that distortion products fall off exponentially with increasing order (2). We therefore approximate the distortion-product amplitudes, A_n, by finding the leading order terms in the expansion. Using just the first two terms to estimate the response at the primary tones, we find in the weak forcing limit ($F_1/F_2 \ll 1$ and $F_1 \ll 1$) that $R_1 \sim F_1$ and $R_2 \sim F_2$. However, in the strong forcing limit ($F_1/F_2 \gg 1$ and $F_1 \gg 1$), we find that $R_1 \sim F_1^{\frac{1}{3}}$ and $R_2 \sim F_1^{-\frac{2}{3}}$.

Adding all contributions to the first distortion product, we find

$$R_1^4 R_2^2 = [\omega_1 - \Delta\omega - \omega_0]^2 A_1^2 + (2R_1^2 + 2R_2^2 + A_1^2 - \mu A_1)^2 A_1^2, \quad [15]$$

which results in the scaling relationship,

$$A_1 \sim \frac{R_1^2 R_2}{R_1^2 + R_2^2}. \quad [16]$$

Continuing the expansion for higher order terms, we arrive at the recurrence relation,

$$A_{n+1} \sim A_n \frac{R_1 R_2}{R_1^2 + R_2^2} \quad [17]$$

Therefore, in the limit of weak stimulus, we find $A_n \sim F_1^{n+1}$, while in the limit of strong stimulus, $A_n \sim F_1^{-\frac{2}{3}-n}$. These power laws and the corresponding level functions of sensitivity are shown in Fig. 2B-C.

Detection threshold for weak stimulus. We now identify the threshold for reliable signal detection in the generic model of the mosquito's auditory system. For weak stimulus, the response of an autonomous oscillator may exhibit a negligible change in amplitude. Instead, information about the signal can be encoded in the phase of oscillation (3). To quantify the degree of phase locking of the response, $z(t)$, to the external signal of interest, we identify the phases in Fourier space, $\psi(\omega)$, where

$$|\tilde{z}(\omega)|e^{i\psi(\omega)} = \frac{1}{T}\int_{-\frac{T}{2}}^{\frac{T}{2}} e^{-i\omega t} z(t)dt, \tag{18}$$

and T is the observation time. After the decay of the transient response, we compute the phase difference between the signal and the response, averaged over many initial conditions of the detector. We quantify the coherence of these responses by calculating the vector strength,

$$\text{Vector Strength} = \frac{1}{N}\left|\sum_{i=1}^{N} e^{i\psi(\omega)}\right|, \tag{19}$$

where N is the number of random initial conditions and $\psi(\omega)$ is evaluated at either the primary tone or distortion product response frequency. Perfect phase entrainment yields a vector strength of 1, while 0 indicates no phase locking. Partial phase locking, as identified by values between 0 and 1, can be sufficient for signal detection (4).

We compute the vector strength over a range of weak stimulus values, F_f, for all four permutations of control parameters (Fig. S1A). Further, we show the response amplitudes of these systems at the frequencies of interest (Fig. S1B). Together, these metrics allow us to identify the weakest stimulus amplitude each of the oscillators can detect. In Fig. 4, we include only the stimulus levels that resulted in a vector strength of at least 0.5.

Transient time & response speed. To quantify the speed of response, we introduce a female flight tone with an abrupt onset at time $t = 0$. For all systems tested, we find that the moving-average amplitude approaches a constant value,

$$Z_i^{\infty} = \lim_{t\to\infty} \frac{1}{T}\int_{t}^{t+T} |z_i(t)|dt \tag{20}$$

for $i = 1, 2$. The bounds of integration span the characteristic time scale of the slower, second oscillator ($T = \frac{2\pi}{\omega_2}$). We define the transient lifetime, τ_i, of the ith oscillator as the time it takes the system to reach and remain within 1% of Z_i^{∞}. Finally, we define the response speed of the ith oscillator as $\frac{\tau_{sho}}{\tau_i}$, where τ_{sho} is the transient time of a harmonic oscillator with a quality factor equal to that of $z_i(t)$. Thus, the response speed indicates how many times faster the system responds than a linear system of equal quality factor. For systems with $\mu_2 > 0$, weak stimulus ($F_f < 0.1$) did not produce transients in $z_2(t)$, as measured by this method. Rather, the mean value of $|z_2(t)|$ began and remained within 1% of Z_2^{∞}. In these scenarios, we do not report the response speed, as it is infinite by our definition.

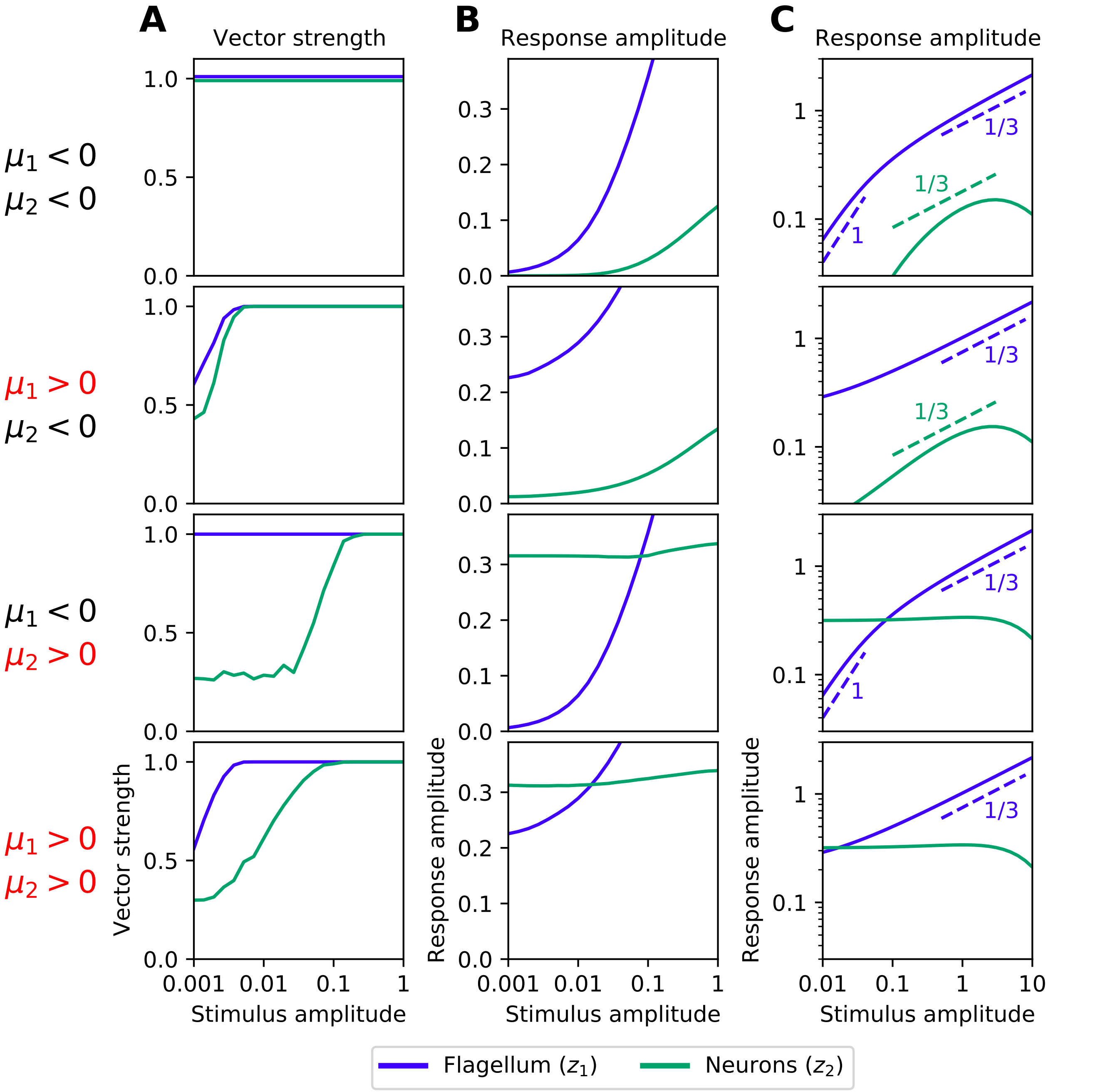

Fig. S1. (**Detection Threshold**) Vector strength (A) and phase-locked amplitude (B) of the responses of the first oscillator at the PT frequency (blue) and the second oscillator at the DP frequency (green) in the composite system. All four permutations of control parameters ($\mu_1 = \pm 0.1$ and $\mu_2 = \pm 0.1$) are shown and identified by the text to the left. (C) Phase-locked amplitude plotted on a logarithmic scale, with dashed lines indicating power-law growth. For all panels, $F_m = 1$, $\omega_f = \omega_1 = 2 \times 2\pi$, and $\omega_m = 3 \times 2\pi$.

References

1. R Stoop, A Kern, Two-Tone Suppression and Combination Tone Generation as Computations Performed by the Hopf Cochlea. *Phys. Rev. Lett.* **93**, 268103 (2004).
2. F Jülicher, D Andor, T Duke, Physical basis of two-tone interference in hearing. *PNAS* **98**, 9080–9085 (2001).
3. L Fredrickson-Hemsing, S Ji, R Bruinsma, D Bozovic, Mode-locking dynamics of hair cells of the inner ear. *Phys. Rev. E* **86**, 021915 (2012).
4. Y Roongthumskul, R Shlomovitz, R Bruinsma, D Bozovic, Phase slips in oscillatory hair bundles. *Phys. Rev. Lett.* **110**, 148103 (2013).
